# A novel locus from the wild allotetraploid rice species *Oryza latifolia* Desv. confers bacterial blight (*Xanthomonas oryzae* pv. *oryzae*) resistance in rice (*O*. *sativa*)

**DOI:** 10.1371/journal.pone.0229155

**Published:** 2020-02-21

**Authors:** Rosalyn B. Angeles-Shim, Junghyun Shim, Ricky B. Vinarao, Ruby S. Lapis, Joshua J. Singleton

**Affiliations:** Plant Breeding Division, International Rice Research Institute, Manila, Philippines; ICAR-Indian Institute of Rice Research, INDIA

## Abstract

Bacterial blight caused by *Xanthomonas oryzae* pv. *oryzae* (*Xoo*) is a major limiting factor to rice productivity worldwide. Genetic control through the identification of novel sources of bacterial blight resistance and their utilization in resistance breeding remains the most effective and economical strategy to manage the disease. Here we report the identification of a novel locus from the wild *Oryza* species, *Oryza latifolia*, conferring a race-specific resistance to Philippine *Xoo* race 9A (PXO339). The locus was identified from two introgression lines i.e. WH12-2252 and WH12-2256 that segregated from *O*. *latifolia* monosomic alien addition lines (MAALs). The discrete segregation ratio of susceptible and resistant phenotypes in the F_2_ (χ^2^_[3:1]_ = 0.22 at p>0.05) and F_3_ (χ^2^_[3:1]_ = 0.36 at p>0.05) populations indicates that PXO339 resistance in the MAAL-derived introgression lines (MDILs) is controlled by a single, recessive gene. Genotyping of a total of 216 F_2_, 1130 F_3_ and 288 F_4_ plants derived from crossing either of the MDILs with the recurrent parent used to generate the MAALs narrowed the candidate region to a 1,817 kb locus that extends from 10,425 to 12,266 kb in chromosome 12. Putative candidate genes that were identified by data mining and comparative sequence analysis can provide targets for further studies on mapping and cloning of the causal gene for PXO339 resistance in the MDILs. To our knowledge, this is the first report of a genetic locus from the allotetraploid wild rice, *O*. *latifolia* conferring race-specific resistance to bacterial blight.

## Introduction

Bacterial blight of rice (*Oryza sativa* L.) caused by *Xanthomonas oryzae* pv. *oryzae* (*Xoo*) is one of the most destructive diseases that negatively impacts the major rice growing regions worldwide. Depending on the onset of the bacterial infection, the degree of susceptibility of the planted cultivar, the virulence of the pathogen and the environmental conditions, the disease can cause significant yield losses of up to 20–81% [1–3].

Traditionally, application of copper compounds or antibiotics has been sufficient in providing rice plants with a degree of protection against the causal pathogen [4, 5]. The intensification of rice monocultures however, have spurred the emergence of new, more virulent races of *Xoo*, rendering most chemical means of disease management largely ineffective. A more efficient, economical and ecologically friendly alternative to control the disease is to provide plants with innate resistance mechanisms that can overcome pathogenic infection. Critical to the success of this approach is the screening of various germplasm to identify sources of novel genetic loci regulating host plant resistance, as well as the development of an efficient strategy to transfer target loci across genomes.

To date, more than 40 genes/loci conferring resistance to bacterial blight have been identified in rice [6], although only eleven (*Xa1*, *Xa3*/*Xa26*, *Xa4*, *xa5*, *Xa10*, *xa13*, *Xa21*, *Xa*23, *xa25*, *Xa27* and *xa41*) have been cloned and functionally characterized [1, 7, 8]. In most cases, the introduction of a single resistance gene/locus into the genetic background of a susceptible cultivar significantly improved rice production and minimized yield losses in regions heavily infected with the pathogen [9–12]. Unfortunately, the extended deployment of rice cultivars with improved bacterial blight resistance over wide areas of cultivation also encouraged the co-evolution of the pathogen, resulting in the eventual breakdown of host plant resistance [12–14]. This re-directed breeding programs towards pyramiding two or more resistance genes/loci in a single genetic background, a strategy that has been proven effective in broadening the spectrum of host plant resistance and enhancing its durability [12, 15–17]. To ensure an upper hand in the continuous evolutionary arms race between plant and pathogen, it is of paramount importance that novel sources of bacterial blight resistance are identified and incorporated into rice breeding programs aimed at improving this valuable target trait.

The wild relatives of rice have long been recognized as a rich reservoir of novel genes underlying important agronomic traits, including tolerance to abiotic challenges, as well as resistance to a wide range of pests and diseases [18–21]. Among the more than 40 bacterial blight resistance genes/loci that have been identified from various germplasm, ten were from wild rice species including *Xa21* from *O*. *longistaminata* [22, 23]; *Xa23* from *O*. *rufipogon* [24]; *Xa27* and *Xa35* from *O*. *minuta* [25, 26]; *Xa29* from *O*. *officinalis* [27]; *Xa30*, *Xa33* and *Xa38* from *O*. *nivara* [28, 29]; *Xa32* from *O*. *australianesis* [30]; and *Xa41(t)* from *O*. *barthii* and *O*. *glaberrima* [31]. Of these genes/loci, *Xa21* which encodes a receptor-like kinase protein and confers a broad-spectrum resistance to *Xoo* races from South and Southeast Asia, has been the most utilized in breeding programs. Improved cultivars with *Xa21*, alone or in combination with other *Xa* genes, have been released and widely cultivated in the Philippines, India, China and Thailand [17, 32, 33].

*O*. *latifolia* is a wild allotetraploid (CCDD) relative of rice that is native to South and Central America [34]. Aside from its high biomass production and lodging resistance, *O*. *latifolia* has also been reported to be an important source of resistance to brown planthopper (*Nilaparvata lugens*), white-backed planthopper (*Sogatella furcifera*), blast (*Magnaporthe grisea*), as well as bacterial blight [20, 21].

In a previous study, we identified two introgression lines i.e. WH12-2255 and WH12-2256 that were derived from monosomic alien addition lines (MAALs) of *O*. *latifolia* with resistance to Philippine *Xoo* races 5 (PXO112), 7 (PXO145), 8 (PXO280) and 9A (PXO339). The resistance of both introgression lines to PXO112, PXO145 and PXO280 was attributed to the genetic contribution of the recurrent parent used to develop the MAALs whereas, resistance to PXO339 was associated with a putative locus within the 13,960 kb alien introgression in chromosome 12 [20]. In this study, we report the identification of a new locus from *O*. *latifolia* regulating the race-specific resistance of WH12-2255 and WH12-2256 to Philippine *Xoo* race 9A. Candidate genes possibly regulating the trait are proposed based on data mining and comparative sequence analysis. To the best of our knowledge, this is the first report of a bacterial blight resistance locus identified from the allotetraploid wild rice, *O*. *latifolia*.

## Materials and methods

### Plant materials and development of mapping populations

**Fig 1:**
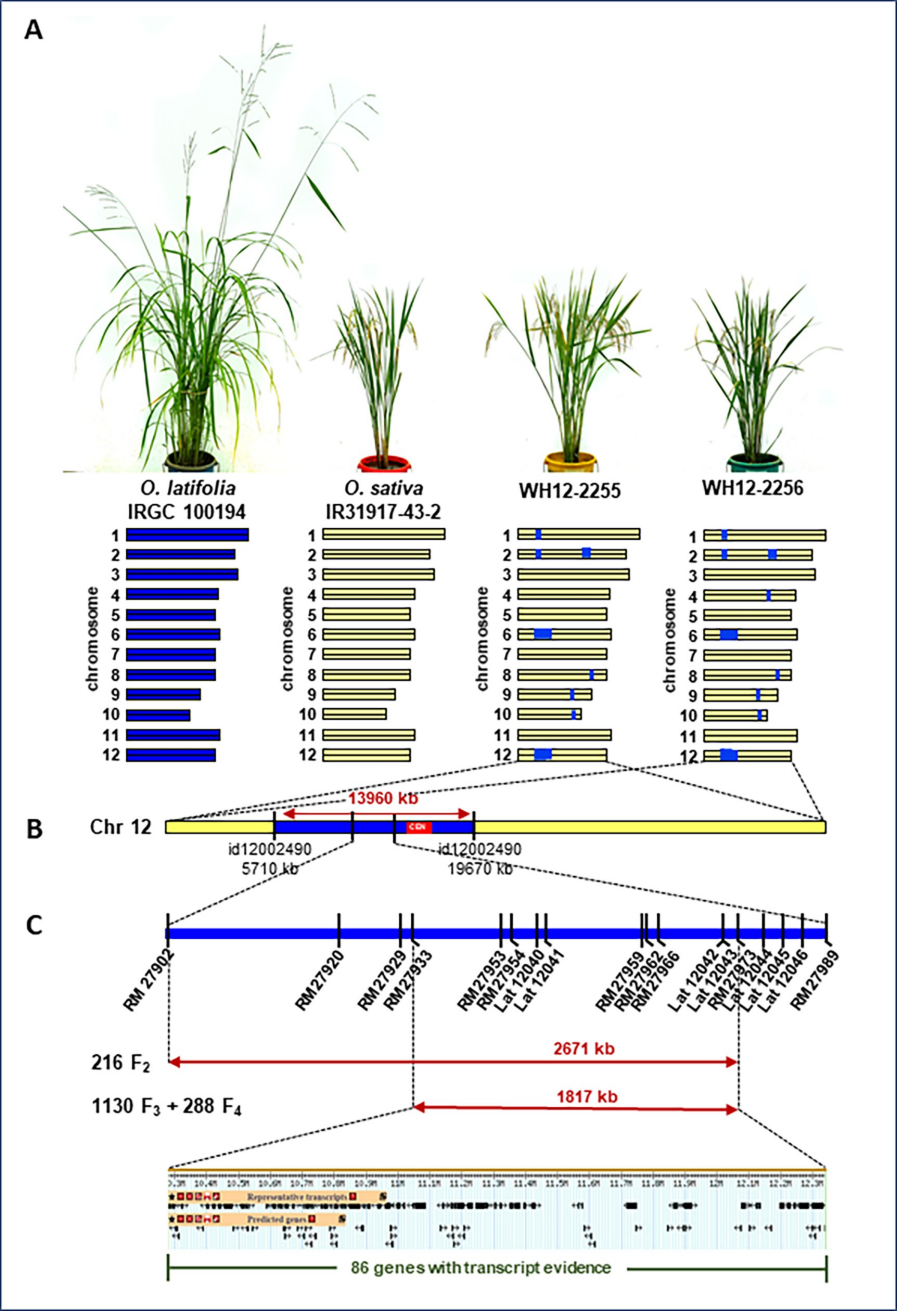
Experimental materials and linkage mapping of PXO339 resistance. (A) Gross morphology and graphical genotype of the experimental materials used in the study. (B) Detailed view of the *O*. *latifolia* introgression in chromosome 12 of the MDILs that is associated with a putative locus conferring PXO339 resistance. (C) Linkage mapping of the candidate locus regulating PXO339 resistance in segregating populations derived from crosses between either of the MDILs and IR31917-43-2. Double-headed arrows indicate the candidate region identified using different segregating populations. Illustration of representative transcripts and predicted genes within the candidate region was generated from [37]. id = SNP marker; RM = SSR marker; Lat = indel marker. Figure not drawn to scale.

Two introgression lines i.e. WH12-2255 and WH12-2256 that segregated from *O*. *latifolia* (IRGC 100914) MAALs in the background of the elite rice breeding line IR31917-42-3 were used in the study. Both lines possess similar *O*. *latifolia* introgressions in chromosomes 1, 2, 6, 8, 9, 10 and 12, with WH12-2256 having an additional alien introgression in chromosome 4 (Fig 1A, Fig 1B). Screening of the MAAL-derived introgression lines (MDILs) against fourteen *Xoo* races from the Philippines showed that WH12-2255 and WH12-2256 are resistant to PXO112, PXO145, PXO280 and PXO339. Marker-trait associations established the presence of a putative locus within the 13,960 kb of the *O*. *latifolia* introgression in chromosome 12 of WH12-2255 and WH12-2256 that confers resistance to PXO339 [20]. To identify the locus regulating the resistance of the two MDILs to PXO339, mapping populations were generated from F_1_ plants developed using either WH12-2255 or WH12-2256 as the female parent and the susceptible IR31917-42-3 as the male parent. The true hybridity of the F_1_s was confirmed by genotyping using two SSR and two CCDD genome-specific indel markers with targets in chromosome 1, 4, 6 and 12 (S1 Table).

### Screening of MDILs and segregating populations for resistance to *Xoo* race PXO339

The reaction of all the experimental materials to PXO339 was determined following the leaf clipping method by Kauffman et al. [35]. Briefly, 21-day-old seedlings were transplanted and maintained in concrete beds inside a screenhouse at the International Rice Research Institute in Los Baños, Laguna, Philippines. At the maximum tillering stage (approximately 45 days after seeding), the tips of 6–10 fully expanded leaves of each plant were cut with scissors dipped in PXO339 suspension (10^9^ cells/ml). Lesion length was measured from three infected leaves per plant after 14 and 21 days of bacterial inoculation, although the reaction of all experimental materials to the pathogen was scored based only on lesion length 21 days after infection. Disease reaction of each plant based on lesion length was scored as follows: <3 cm = resistant, 3.1–5 cm = moderately resistance and >3 cm = susceptible [15]. The rice cultivar IR24 was used as the susceptible control, whereas IRBB21 was used as the resistant control throughout the course of diseases resistance evaluation.

### Linkage analysis of PXO339 resistance locus from *O*. *latifolia*

Two-hundred sixteen F_2_ plants, as well as 1,130 F_3_ and 288 F_4_ plants derived from F_2_ and F_3_ lines with informative recombination points, respectively, were genotyped using rice SSR markers, as well as CCDD genome-specific indel markers designed based on the available genome sequence of *O*. *alta* (IRGC 105143) (Table 1). All the markers used were selected or designed to amplify targets within the 13,960 kb *O*. *latifolia* introgression in chromosome 12 of both MDILs. Genomic DNA was extracted from the leaf tissue of all the experimental materials using the TPS method. Briefly, 2 cm leaf tissues were homogenized in TPS buffer (100 mM Tris-HCl (pH 8.0), 1 M KCl, 10 mM EDTA) using the 2010 Geno/Grinder® (NJ, USA). After centrifugation, the supernatant was recovered, mixed with an equal volume of isopropyl alcohol and centrifuged to precipitate the genomic DNA. The DNA pellets were then washed with 70% ethanol, dried and re-suspended in TE buffer (10mM Tris-HCl (pH 8.0); 1mM EDTA) with RNase A (10mg/ml). SSR and indel targets were amplified from the extracted DNA samples following standard PCR protocol [36]. Amplicons were resolved in 3% agarose gel in 1X Tris-Borate-EDTA buffer.

The genetic inheritance of POX339 resistance was determined based on phenotypic segregation ratio in the F_2_ and F_3_ populations, and was validated using the chi-square (χ^2^) test.

**Table 1:** SSR and indel markers used to genotype mapping populations derived from *O*. *latifolia* MDILs. All markers target the 13.96 Mb *O*. *latifolia* introgression in chromosome 12 of the MDILs.

No.	Primer Name	Marker type	Forward primer	Reverse primer	Map position (kb)
1	Lat 12002	indel	TCCATCATCGGTATCGATG	GTGGAGATCAAATCAATGTGG	5101
2	Lat 12003	Indel	TCTCCCCAGCTCCATTATGC	GCTGTCACAGCATTTGTAGCA	7485
3	Lat 12035	indel	CTCGAAAGCCGTCATGAAC	CCAACAACAGGAGCTTCAC	9570
4	RM27902	SSR	TATTCGTCGTCGTCTCGTCATCC	GTTGACGTTGACATTTGCAGTGG	9595
5	RM27920	SSR	AAAGCGAGAAATCCGGAGATGG	TCCTCTCTCAAATCTCCTCGAAGC	10139
6	RM27929	SSR	TGGCCAACTCGCTAGATCTCTTGG	ACCCTCGTCATCAAATCCGTTGC	10319
7	RM27933	SSR	TCCTCTGTCATATGGCTGTAAACG	GGACAAGGAGGAACTATTGATTGG	10425
8	RM27953	SSR	CACCTGGCTCCTCATCAAGTACC	TGGCTTAGTCTTGTGGAGACACG	11225
9	RM27954	SSR	CATATCCGCATTTGAGTCACTTCG	AACGCCAACTATGGAACTGTTTCG	11262
10	Lat 12040	indel	AGGTTATAGGCATTGGCTGG	ATTCAGATACCATCACACTC	11352
11	Lat 12041	indel	GATGTGTCAAGGAAGCTGTG	GCAAGCCATAATCATCAGAG	11376
12	RM27959	SSR	TGGAGATGGGTGGACGGTTAGG	ATTTAGCGAAGCGGTTGGTAATGACG	11902
13	RM27962	SSR	GGGAGTCGTGGATTCTGAGACG	ATCCCACGCCAGGAGATAATAAGG	12080
14	RM27966	SSR	TCTGAGCCAACAGTAAGAGTCAGG	TGTCACCCGTAGTGTTTGTACGG	12120
15	Lat 12042	indel	TGCAGCTATGATCACATGGC	TCCTTGAAGCTTGTCCTGTG	12242
16	Lat 12043	indel	ACCATCAATCTCAAAGCAAC	AATGAAGGCTTTCTGATGTG	12243
17	RM27973	SSR	CCACACTGCCCAGGATTTAAGC	CTGTTCCCATCATCCAAATGACC	12266
18	Lat 12044	indel	TTTGAAGAGGAGTGGGACAC	CAAAAGGGAAGCATGCAACC	12326
19	Lat 12045	indel	GGTGGACTGATTAGAGTGCT	GGATAGTCTGGTAAGCTAGC	12430
20	Lat 12046	indel	CTACGGACTACGGTACATTC	CGCAGAGATGTTGAGACTTG	12689
21	RM27989	SSR	GAGGGCTCCCTGACAACAACC	CGTCGGCCGTGTCATAGTGG	12842
22	RM28038	SSR	ATGAACGATGAGGGTTCAAAGG	ATCTGTCGTCCTGATGTACTTTGC	13738
23	RM28048	SSR	TTCAGCCGATCCATTCAATTCC	GCTATTGGCCGGAAAGTAGTTAGC	14106
24	RM28075	SSR	GGGACTTGGGACCAGTTTATGG	TCAGGTCTGTTGGATTCCATGC	15007
25	RM28254	SSR	ACCCTGACTTATGACTTGTTCG	CTCAGTGTTTGAAGATCAGTGG	19062
26	RM28277	SSR	TGCACCACCTATTTCAATCCACTCC	CCTTCCTCAAGGGAAATCACAGAAGC	19426

### Putative gene prediction and comparative sequence analysis

Details of the annotated genes within the defined candidate region including gene IDs, description/function and ontology were retrieved from the Rice Annotation Project Database [37]. Possible candidates regulating PXO339 resistance were identified based on the reported functions of each gene within the region of interest. Comparative sequence analysis of the identified putative genes in IR31917-42-3, WH12-2255, WH12-2256 and Minghui 63 was carried out to identify causal mutations resulting in the phenotypic response of the experimental materials to PXO339 infection. Minghui 63 is an elite fertility restorer line that has been identified to have *xa25*, a recessive gene conferring a similar race-specific resistance to PXO339 [38, 39]. Comparative sequence analysis of *xa25* was carried out among the test materials to determine whether the locus regulating the resistance of the MDILs to PXO339 was conferred by *xa25* or by a novel resistance locus.

Whole genome sequencing using the NovaSeq 6000 platform was outsourced to Macrogen Inc., South Korea using genomic DNA from young leaves that was extracted following a modified CTAB method [40]. Assembly of the whole genome sequence data for each line was guided by the rice cv. Nipponbare reference genome [37] using ABySS, a de novo sequence assembler intended for short paired-end reads and large genomes [41]. Comparative genome sequence analysis of the 1,817 kb candidate region in chromosome 12 was carried out using the BioEdit Sequence Alignment Editor [42].

## Results and discussion

### Reaction of experimental materials to PXO339

**Fig 2:**
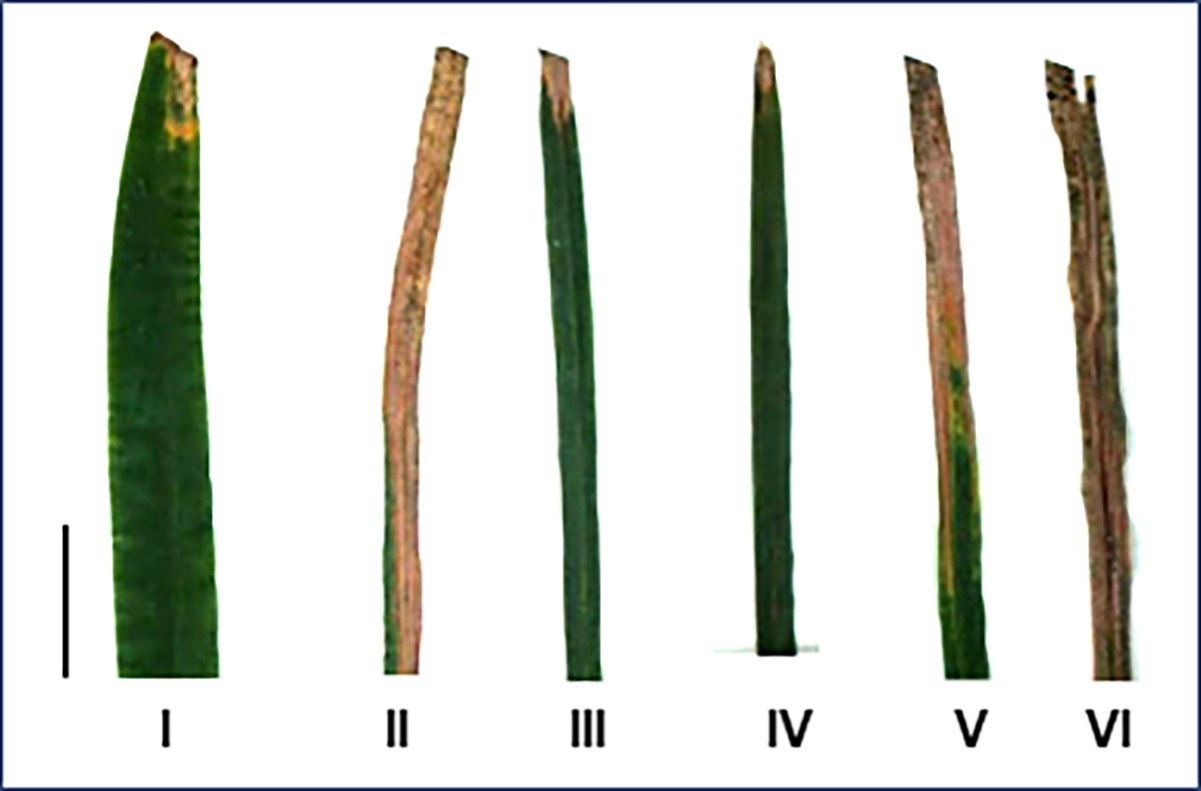
Resistance reaction of the experimental materials to PXO339. Lesion length in *O*. *latifolia* (I), IR31917-42-3 (II), WH12-2255 (III), WH12-2256 (IV), WH12-2255 x IR31917-42-3 F_1_ (V) and WH12-2256 x IR31917-42-3 F_1_ (VI) leaves 21 days after PXO339 inoculation, with I, III and IV exhibiting lesions shorter than 3 cm. bar = 5cm.

The elite breeding line IR31917-42-3 exhibited leaf lesions averaging 10.50 cm, whereas *O*. *latifolia*, WH12-2255 and WH12-2256 had average lesion lengths of >3 cm after 21 days of inoculation with the Philippine *Xoo* race 9A (Fig 2). These results are in agreement with the previously reported reactions of the same experimental materials to the pathogen. The race-specific resistance of WH12-2255 and WH12-2256 has been associated with a putative locus within the *O*. *latifolia* introgression in both MDILs that extends from 5,710 to 19,670 kb in chromosome 12 and that includes the centromeric region [20].

The F_1_ plants developed from crosses between either of the MDILs and IR31917-42-3 were all susceptible to PXO339, with leaf lesions measuring more than 15 cm on the average after 21 days of pathogen inoculation (Fig 2). The reaction of the F_1_s suggest a recessive mode of inheritance of PXO339 resistance in both MDILs. All F_1_ plants used for resistance screening were confirmed as true hybrids by genotyping using select SSR and indel markers.

### Inheritance of PXO339 resistance in the mapping populations

**Fig 3:**
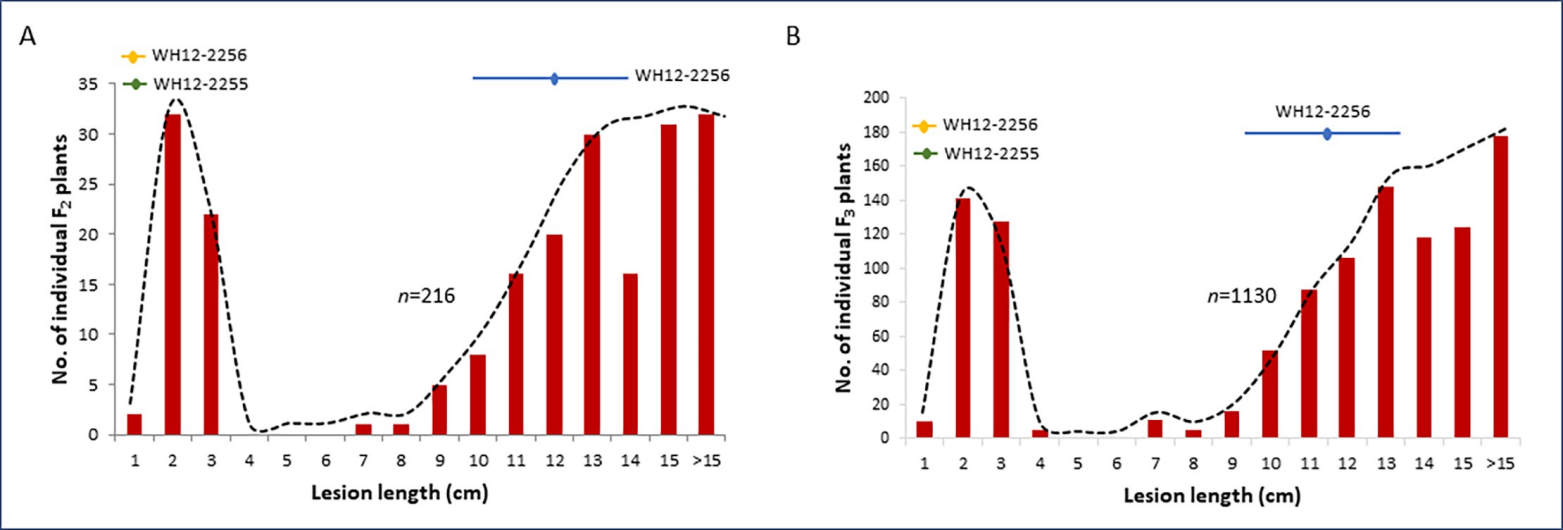
Phenotype distribution in segregating populations. Lesion length in the leaves of F_2_ (A) and F_3_ (B) plants 21 days after PXO339 inoculation showing a bimodal distribution. Broken lines highlight the separated distribution of resistant and susceptible phenotypes in both populations. Horizontal lines indicate the range of lesion length observed in the parental lines. n = the number of individual plants in each population.

The distribution of lesion length in response to PXO339 inoculation was bimodal in both the F_2_ and F_3_ populations, with a clear gap at 4–6 cm in the F_2_ and at 5–6 cm in the F_3_ population (Fig 3). Based on lesion length, the F_2_ plants segregated into 56 resistant to 160 susceptible, whereas the F_3_ plants segregated into 279 resistant to 851 susceptible. Both ratios fit the phenotypic segregation pattern for a recessively inherited trait. Chi-square analysis of the observed versus the expected Mendelian ratio for susceptible and resistant phenotypes for both the F_2_ (χ^2^_[3:1]_ = 0.22 at p>0.05) and F_3_ populations (χ^2^_[3:1]_ = 0.36 at p>0.05), combined with the observed reaction of the F_1_ hybrids to PXO339, established the monogenic and recessive inheritance of the trait.

### Linkage analysis of MDIL resistance to PXO3399 and candidate gene prediction

Genotyping 216 F_2_ plants using a total of 26 SSR and indel markers targeting the wild introgression in chromosome 12 of both MDILs identified the co-segregation of the indel marker, Lat 12043 with PXO339 resistance. Lat 12043 maps close to the centromere at 12,243 kb in the short arm of chromosome 12. Fine mapping using the F_2_ population narrowed the candidate gene region to a 2,671 kb locus bound by the SSR markers RM27902 at 9,595 kb and RM27973 at 12,266 kb. Genotyping of an additional 1130 F_3_ and 288 F_4_ plants further narrowed the candidate gene region to a 1,817 kb locus that is flanked by RM 27933 at 10,425 kb and RM27973 at 12,266 kb (Fig 1C, Table 1). Database mining identified 86 genes with open reading frames within the region of interest, with 17 of the genes having alternative transcripts or splice isoforms (S2 Table). Of the 86 gene annotations, 45 translates into known or specific proteins, whereas the rest code for hypothetical proteins and non-protein coding transcripts. Of these, 20 genes have been identified to have molecular and/or biological functions and/or to code for cellular components, whereas the remaining 25 genes have unknown functional representation based on gene ontology analysis. Among the 45 genes with known protein products, only nine have been reported to be involved in various defense response networks in various crops. These include *Os12t0278800* (similar to CCCH domain containing zinc finger), *Os12t0280050* (similar to thioredoxin family protein), *Os12t0281300* (Pi-ta protein; nucleotide-binding site (NBS)-leucine-rich repeat (LRR)), *Os12t0281600* (NB-ARC domain containing protein), *Os12t0283400* (pectinesterase inhibitor domain-containing protein), *Os12t0286300* (cytochrome P450 domain containing protein), *Os12t0290100* (similar to protein kinase), *Os12t0299650* (Myb/SANT-like domain containing protein) and *Os12t0405700* (similar to wound-induced basic protein). Previous reports show that orthologous protein products of *Os12t0278800*, *Os12t0280050*, *Os12t0280050*, *Os12t0283400*, *Os12t0286300*, *Os12t0290100*, *Os12t0299650* and *Os12t0290100* elicit quantitative resistance against fungal and bacterial pathogens in cotton [43–45, 46], barley [47], potato [48], soybean [49], wheat [50, 51], grapes [52] and *Arabidopsis* [53]. Quantitative resistance is usually non-race-specific and is controlled by quantitative trait loci (QTL) or multiple genes. These QTLs have small effects that confer partial but durable resistance to most races of the same pathogen [54, 55]. Although quantitative resistance may be mediated by similar genes involved in a qualitative defense response, the predominant mechanisms of quantitative resistance extends beyond pathogen recognition and may include specific defense-related outputs such as strengthening of the cell wall and biosynthesis of defense compounds.

In contrast, qualitative host resistance is often defined by race-specificity and monogenic inheritance of a dominant or recessive resistance (R) gene [56]. The R genes encode proteins that can directly or indirectly recognize avirulence (Avr) effectors that are delivered by pathogens into the plant cells. Pairwise associations between the R proteins and the Avr effectors usually trigger a defense response that is characterized by rapid cell death in areas surrounding an infection. This kind of hypersensitive response effectively restricts the spread of the disease in other parts of the plant [57]. Of the nine genes identified in this study to have defense response function, only *Os12t0281300*, *Os12t0281600* and *Os12t0405700* encode proteins that have been reported to confer qualitative resistance to pathogen attacks [58–61].

In the present study, the discrete segregation of phenotypes in the F_2_ and F_3_ mapping populations clearly established PXO339 resistance of the *O*. *latifolia* MDILs as a qualitative trait, making *Os12t0281300*, *Os12t0281600* and *Os12t0405700* the most probable candidates for the resistance locus. *Os12t0281300* which encodes *Pi-ta* has only been associated with resistance to rice blast [46] but not to bacterial blight. Comparative sequence analysis of *Pi-ta* in WH12-2255 and WH12-2256 relative to the susceptible IR31917-42-3 showed mutations in the promoter and coding regions, as well as in the 3’ and 5’UTR of the gene (S1A Fig). None of the mutations however, induced changes in the translated protein product, eliminating the possibility of this gene regulating PXO339 resistance in the MDILs. Similarly, genetic variations in the form of SNPs and/or single base insertion were also identified in the promoter and coding region of *Os12t0281600* sequence from WH12-2255 and WH12-2256 relative to IR31917-42-3 (S1B Fig). Again, none of the mutations resulted in changes in the translated protein products, excluding the possibility of this gene controlling PXO339 resistance. In contrast, a 9-base insertion and three other SNP variations in the promoter region, as well as a single SNP variation in the 3’UTR was observed in the wound-induced basic protein gene of both MDILs but not in IR31917-43-2. The 9-base insertion generated an additional E-box motif upstream of the putative core promoter (S1C Fig, i and ii). Pathogen-inducible plant promoters contain multiple cis-acting elements that regulate gene expression. The additional E-box motif in the promoter region of *Os12t0405700* could possibly enhance the expression of the gene during pathogen attack, thereby conferring PXO339 resistance in both MDILs. At this point, the proposed effects of the additional E-box on the expression of the wound-induced basic protein is purely speculative and would require validation through further studies.

### PXO339 resistance of *O*. *latifolia* MDILs is not due to *xa25*

The recessive *xa25* (*LOC_Os1229220*/O*s12g0476200*) which encodes a nodulin MtN3 family protein is a race-specific R gene that was identified from Minghui 63 to confer resistance to PXO339 [38, 39]. The gene maps at 17,302 kb near the centromere of chromosome 12. Although the alien introgression in WH12-2255 and WH12-2256 originally covers the physical position of *xa25* in chromosome 12, mapping of the candidate locus regulating PXO339 resistance in both MDILs was narrowed down to a 1,817 kb region flanked by the markers RM 27933 at 10,425 kb and RM 27973 at 12,266 kb. This physical interval is well outside the location of *xa25* locus, indicating a novel gene/locus regulating PXO339 resistance in the MDILs. Comparative sequence analysis showed a 6-bp deletion (851_856delCCGCCG) in the coding region of *xa25* locus in WH12-2255 and WH12-2256, and a 3-bp deletion (739_741delCGC) in exon 6 of the same gene in IR31917-43-2 relative to Minghui 63, further suggesting the presence of a different locus regulating PXO339 resistance in WH12-2255 and WH12-2256. Analysis of the promoter region for variations in the sequence of the effector binding elements (EBE) [62] in the experimental materials showed a T>A mutation at -225 bp from the transcription start site of IR31917-43-2, WH12-2255 and WH12-2256 relative to Minghui 63. At -248 bp from the transcription start site, a G>A mutation in WH12-2255 and WH12-2256, and a G>C in IR31917 relative to Minghui 63 were also identified (S2 Fig). EBEs serve as recognition sites for transcription activator-like effectors (TALEs) that are delivered by *Xoo* into host plants. In Minghui 63, nucleotide polymorphism in the EBE of the recessive *xa25* compared to that of the dominant allele prevents the nucleotide-specific binding of TALEs and results in a race-specific *Xoo* resistance of the plant [38, 62, 63]. The observed nucleotide variations in the EBEs of both MDILs and IR31917-42-3 relative to Minghui 63 further support the results of the study, indicating that a novel locus, different from *xa25*, from *O*. *latifolia* is regulating the resistance of WH12-2255 and WH12-2256 to PXO339.

## Conclusion

Bacterial blight is a destructive disease that can cause extensive yield losses in rice. To date, identification and introgression of genes conferring resistance to bacterial blight remain the most effective and economical means of controlling the disease. Given the impermanence of bacterial blight resistance in cultivars bred to have them and the continuous emergence of new and more virulent races of the pathogen, identification of new sources of resistance that can be integrated into breeding programs will be crucial in our efforts to gain the upper hand in the arms race between plant and pathogen.

In this study, we identified a novel locus from two introgression lines derived from *O*. *latifolia* MAALs that confers resistance to bacterial blight race PXO339. The locus was delimited to an 1,817 kb introgression from chromosome 12 of *O*. *latifolia* and is inherited in a recessive, Mendelian fashion. Putative genes that were determined to possibly regulate PXO339 resistance in the MDILs provide targets for further studies on the identification and cloning of the causal gene responsible for the trait.

By and large, the results of this study highlights the importance of wild rice relatives, as well as exotic germplasm generated from wild rices as sources of novel genetic variation to improve trait performance in the crop. The natural genetic variation found in wild *Oryza* species is a product of strong selective pressures that allowed the evolution of adaptive mechanisms against a multitude of biotic and abiotic stresses. More often than not, these mechanisms are exclusive only to the wild rices and cannot be found in their cultivated counterparts, as in the case of PXO339 resistance from *O*. *latifolia*. To the best of our knowledge, this is the first report of a bacterial blight resistance locus identified from the wild rice species, *O*. *latifolia*.

## Supporting information

S1 Fig Structure of *Os12t0281300*, *Os12t0281600* and *Os12t0405700*. (A) Gene model for *Os12t0281300* in WH12-2255 and WH12-2256 showing SNP mutations in the promoter, 3’ and 5’UTR, and exons. (B) Gene model for *Os12t0281600* in WH12-2255 and WH12-2256 showing SNP mutations and a base insertion in the promoter region and SNP mutations in the exon. (C) Gene model for *Os12t0405700* in WH12-2255 and WH12-2256 showing mutations in the promoter region as well as in the 3’untranslated region. (i) A 9-bp insertion (black text) in the promoter region of the gene resulting in an additional E-box motif (underlined black text). Text highlighted in green indicate the putative transcription start site.
(TIF)

S2 Fig Nucleotide polymorphism in the reported effector binding elements (EBE) of *xa25*/*Xa25* of WH12-2255, WH-1256, IR31917 and Minghui 63. Yellow boxes highlight sequence variations in the EBE. Scale indicates position of bases from the transcriptional start site.
(TIF)

S1 Table SSR and CCDD genome-specific markers used to confirm the hybridity of the F_1_s. (XLSX)

S2 Table Annotations of genes within the 1817 kb candidate region in chromosome 12. (XLS)
