# Supplementary figures and images for: A novel locus from the wild allotetraploid rice species Oryza latifolia Desv. confers bacterial blight (Xanthomonas oryzae pv. oryzae) resistance in rice (O. sativa)
Source: PLoS One. 2020 Feb 21;15(2):e0229155. doi: 10.1371/journal.pone.0229155 (PMC7034821; doi:10.1371/journal.pone.0229155)

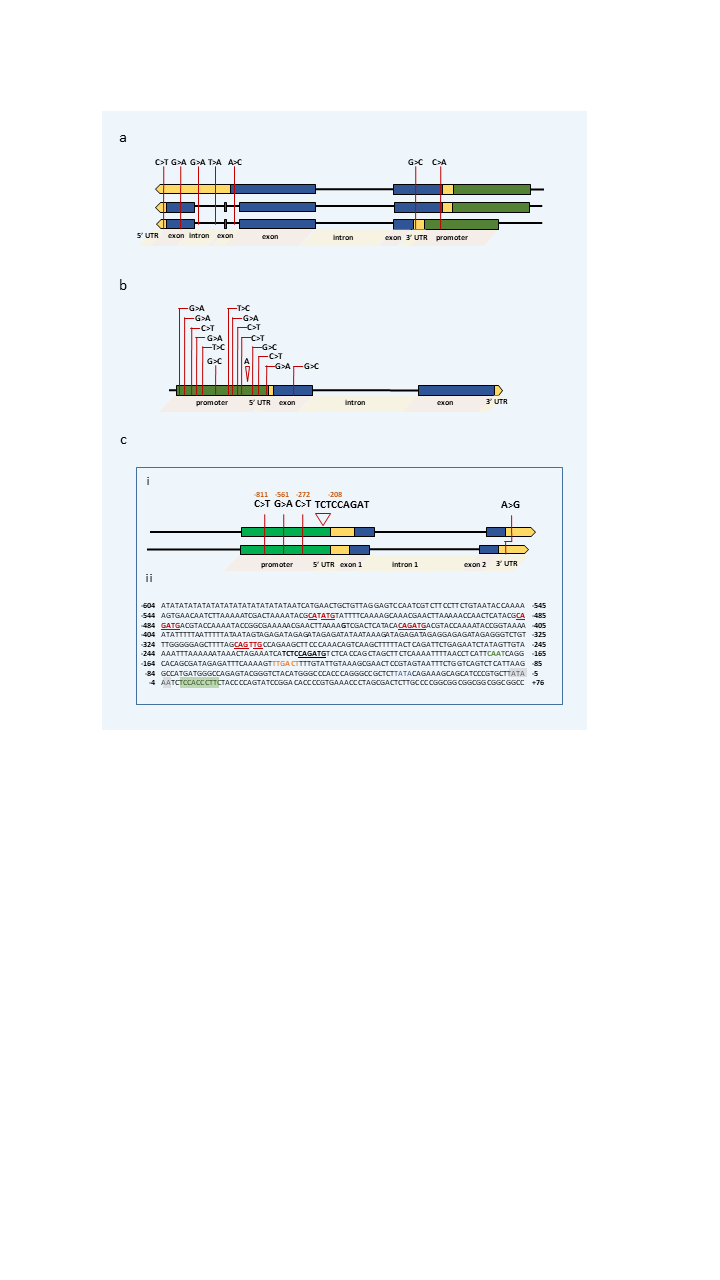

Supplement: S1 Fig — (A) Gene model for Os12t0281300 in WH12-2255 and WH12-2256 showing SNP mutations in the promoter, 3’ and 5’UTR, and exons. (B) Gene model for Os12t0281600 in WH12-2255 and WH12-2256 showing SNP mutations and a base insertion in the promoter region and SNP mutations in the exon. (C) Gene model for Os12t0405700 in WH12-2255 and WH12-2256 showing mutations in the promoter region as well as in the 3’untranslated region. (i) A 9-bp insertion (black text) in the promoter region of the gene resulting in an additional E-box motif (underlined black text). Text highlighted in green indicate the putative transcription start site. (TIF) [file pone.0229155.s001.tif]

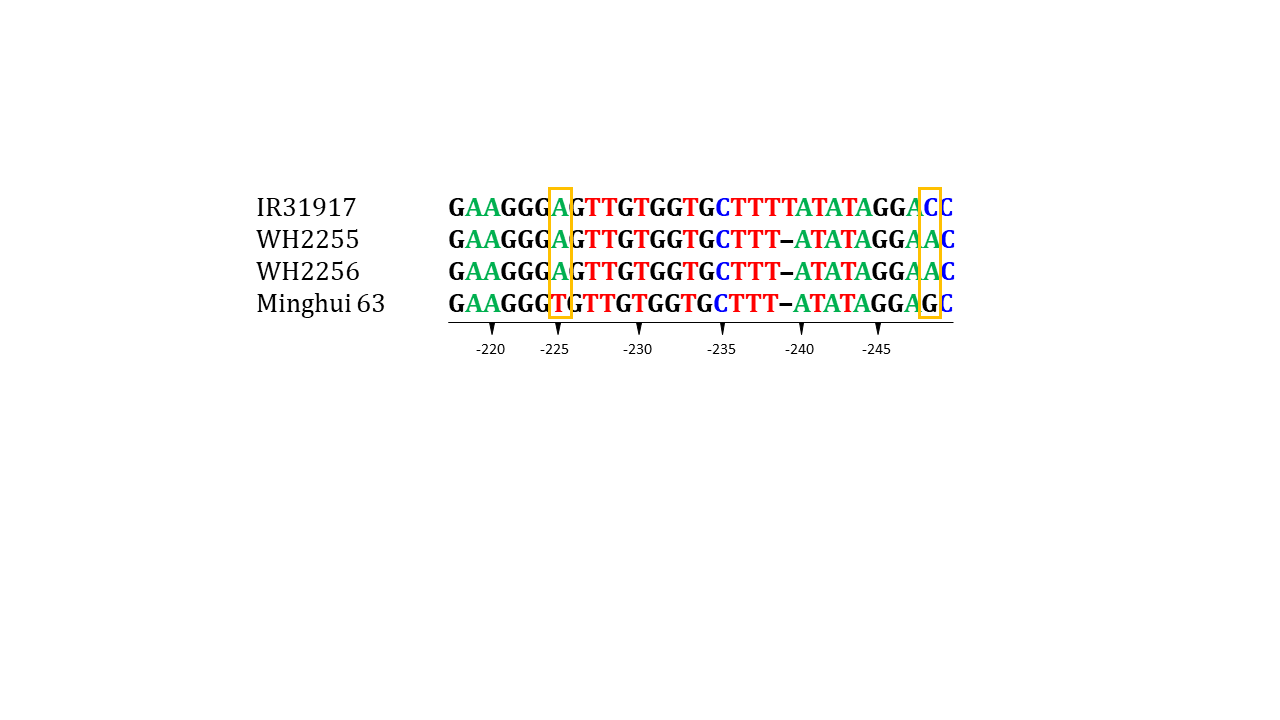

Supplement: S2 Fig — Yellow boxes highlight sequence variations in the EBE. Scale indicates position of bases from the transcriptional start site. (TIF) [file pone.0229155.s002.tif]
